# Analysis of Microstructure of the Cardiac Conduction System Based on Three-Dimensional Confocal Microscopy

**DOI:** 10.1371/journal.pone.0164093

**Published:** 2016-10-07

**Authors:** Daniel Romero, Oscar Camara, Frank Sachse, Rafael Sebastian

**Affiliations:** 1 Grupo de Investigacion e Innovacion Biomedica, Instituto Tecnologico Metropolitano, Medellin, Colombia; 2 Physense, Dept. of Information and Communication Technologies, Universitat Pompeu Fabra, Barcelona, Spain; 3 Cardiovascular Research and Training Institute and Bioengineering Department, University of Utah, Salt Lake City, Utah, United States of America; 4 CoMMLab, Dept. of Computer Sciences, Universitat de Valencia, Valencia, Spain; University of Minnesota, UNITED STATES

## Abstract

The specialised conducting tissues present in the ventricles are responsible for the fast distribution of the electrical impulse from the atrio-ventricular node to regions in the subendocardial myocardium. Characterisation of anatomical features of the specialised conducting tissues in the ventricles is highly challenging, in particular its most distal section, which is connected to the working myocardium via Purkinje-myocardial junctions. The goal of this work is to characterise the architecture of the distal section of the Purkinje network by differentiating Purkinje cells from surrounding tissue, performing a segmentation of Purkinje fibres at cellular scale, and mathematically describing its morphology and interconnections. Purkinje cells from rabbit hearts were visualised by confocal microscopy using wheat germ agglutinin labelling. A total of 16 3D stacks including labeled Purkinje cells were collected, and semi-automatically segmented. State-of-the-art graph metrics were applied to estimate regional and global features of the Purkinje network complexity. Two types of cell types, tubular and star-like, were characterised from 3D segmentations. The analysis of 3D imaging data confirms the previously suggested presence of two types of Purkinje-myocardium connections, a 2D interconnection sheet and a funnel one, in which the narrow side of a Purkinje fibre connect progressively to muscle fibres. The complex network analysis of interconnected Purkinje cells showed no small-world connectivity or assortativity properties. These results might help building more realistic computational PK systems at high resolution levels including different cell configurations and shapes. Better knowledge on the organisation of the network might help in understanding the effects that several treatments such as radio-frequency ablation might have when the PK system is disrupted locally.

## Introduction

Efficient pump function of the heart requires synchronised activation and contraction of cardiac tissues. Specialised tissues known as the cardiac conduction system (CCS) are responsible for synchronisation of mechanical activation by fast distribution of the electrical signals that trigger contraction [1]. In the ventricles, the CCS comprises Purkinje (PK) fibres, which are able to conduct electrical signals up to almost an order of magnitude faster than the working myocardium, thus facilitating the synchronous electrical activation of the whole heart [2, 3]. The CCS includes the heart’s primary pacemaker, the sinus node in the right atria, which initiates each beat. Electrical signals are transmitted to the ventricle through the atrio-ventricular (AV) node. A bundle located high in the inter-ventricular septum and beneath the AV node, known as the His bundle, branches into the left and right bundle branches descending sub-endocardially into their corresponding ventricles [4]. These branches spread out forming a complex network referred as to the PK network, which is the distal portion of the CCS, which runs both through the ventricular cavity as free-running Purkinje strands (FRPS) and along the endocardium [5]. Depending on species the PK network is electrically insulated from other surrounding tissue by a sheath of collagen, creating an electrical insulation [6]. Electrical signals are therefore only passed to the working myocardium at terminal points known as Purkinje-ventricular junctions (PVJs) [2, 5, 7]. The coupling between PK cells and working myocytes presents two types of configurations: (i) a funnel connection, with a direct coupling to myocytes and (ii) a sheet interface or resistive barrier, composed of transitional cells (T cells) that serve as an intermediate layer between the PK cells and myocytes [8]. Transitional cells are present at the boundaries of the sinus and atrio-ventricular nodes as they connect to their surrounding tissues [9].

Previous studies about the CCS described qualitatively its structure using 2D imaging techniques based on ink markers [5, 10–14], light microscopy [8], projection microscopy [6], EGFP imaging [15], and optical Imaging [16]. It has only been in recent years that researchers have extracted 3D information about the macro-structure of the PK network. In some studies only the free-running Purkinje strands (FRPS) were analysed using high-resolution ex-vivo MR system [17, 18], while in others the whole proximal 3D structure was reconstructed or simply visualised [3, 14, 19, 20]. In [21] it was attempted a quantitative macroscopic description of the PK network structure performed on 2D photographs of bovine hearts stained with Chinese ink, where branch lengths and furcation angles were measured.

Ono et al. [22] described two types of PK cells, tubular and polygonal, but did not provide information about their different morphological characteristics. Different techniques have been used to provide quantitative measurements on single cell morphology, e.g. lengths, widths, heights, areas, volumes. Single cell isolation of PK cells has been performed in mouse [23], rabbit Cordeiro1998 and dog [24], where 2D measurements were provided. Microscopy studies of PK cell morphology have taken direct measurements of cell dimensions but have used simple ellipsoid models to approximate the 3D characteristics such as surface areas and volumes [22, 25]. With regards to working myocytes, single cell 3D reconstructions have been obtained with confocal microscopy data [26], therefore being able to compute more accurate measures of cell features. In [26] single working myocytes were segmented in 3D and subsequently, volume, length, width and height were estimated. Volumes were calculated as the sum of voxels contained inside the cell and length/width/height estimates were obtained by fitting a bounding box oriented after applying singular value decomposition on second-order central image moments. In [27] stained cells were projected onto a digitised surface and the length and width measured with a sonic digitiser. Volumes were also obtained from rotation of 2D planes along the long axis and assuming that cells have the shape of irregular cylinders. In [28] and [29] the volume of myocytes was calculated using a Coulter Channelyzer system as well as by multiplying the length by the cross-sectional area (obtained from electron micrographs) of isolated cells, obtaining comparable results. To our knowledge these PK cell measurements had not previously been taken from 3D reconstructions.

An important aspect of the PK network involves the electrical coupling between neighbouring cells, which is achieved through gap junction channels. These are channels connecting the cytosol of two cells to allow ion flow. Gap junction channels are made of proteins known as connexins: channels with Cx40 have the largest conductance, Cx43 provides an intermediate conductance, and Cx45 has the lowest conductance in cardiac cells [30]. Although a combination of these proteins and channels is present across different tissues they differ in abundance [9]. The Cx40 is predominantly found in PK fibres, while Cx43 is mainly present in the working myocardium [31–33]. Commonly, gap junctions are preferentially located at cells’ ends (end-to-end connection) to facilitate longitudinal signal propagation among cells while smaller expression is found allowing lateral communication between cells [33, 34].

An improved understanding of the 3D structure of the PK network, and in particular their connection to working myocardial cells, has implications for our knowledge of cardiac diseases [35]. The PK system plays an important role in arrhythmogenesis, for example in macro- and micro-reentries due to unidirectional blockage giving rise to ventricular tachycardia [36]. In radio-frequency ablation therapy for ventricular arrhythmias the targeting of Purkinje-like-potentials (PLPs) at the boundaries of a scar border zone has proven to be an effective method for eliminating the recurrence of ventricular fibrillation [37]. Therefore an increasing number of computational models have incorporated and (in silico) analysed the CCS in order to understand the underlying pathologies and optimize treatment [38–41]. Experimental work [42] and modelling studies [43, 44] on the influence of the PK micro-structure on arrhythmogenesis has focused on the coupling and electrical disturbances of the PVJs.

An accurate 3D structural analysis of the distal portion of the CCS can enhance the possibility of developing realistic generational algorithms of the PK system, as it has been already done at macroscopic scale [21, 45, 46]. The goal of this paper is to obtain a set of quantitative measurements of the 3D microstructure of the PK system at its distal portion in order to better characterise its morphology. Firstly, we provide 3D information on cell dimensions from segmentations of PK fibres obtained from confocal microscopy imaging. Furthermore, we analyse the PK system by modelling it as a 3D graph and then estimating measures of network complexity as well as other measures such as branch lengths and angles at furcations.

## Materials and Methods

### Tissue preparation

For the microscopic characterisation of the PK system, we designed a pipeline to acquire and process tissue samples from rabbit hearts. We applied stored left-over tissues from a study approved by the Institutional Animal Care and Use Committee (Protocol # 09-03015) at the University of Utah. The original samples were obtained from adult rabbits that were anesthetised with pentobarbital and anticoagulated with heparin. Following thoracotomy hearts were quickly excised and placed in a modified oxygenated Tyrod’s solution at room temperature. The hearts were retrogradely perfused. Tyrode’s solution including wheat germ agglutinin (WGA) conjugated to Alexa Fluor 555 (Invitrogen, Carlsbad, CA). Through the hearts, WGA binds to glycocalix of carbohydrates in the cell membrane and extracellular proteins. This method allowed for a homogeneous distribution of the dye throughout the heart. The hearts were subsequently fixed through the same line of the Langendorff perfusion with paraformaldehyde. Biopsies were made from left and right ventricle lateral walls (mid and apical), papillary muscles, and septal wall. Afterwards, the biopsies were stored in paraformaldehyde.

### Confocal Microscopy Imaging

**Fig 1:**
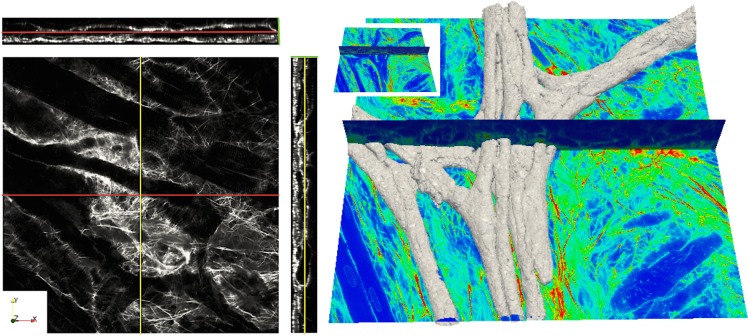
Processed images from 3-D stack of WGA labeled endocardial ventricular tissue from rabbit. The left panel illustrates a sample stack in XY (plane parallel to endocardium), XZ, and YZ views where PK cells show a sparse tubular system (T-system). The right panel shows the corresponding 3D reconstruction of the cells in a stack, superimposed on the confocal microscopic slices. The image stack was deconvolved and corrected for depth-dependent attenuation. Field of view: 318 × 318 *μm*.

Images were obtained within 2 months after tissue preparation. 3D image stacks were acquired from tissue samples in bottom-up direction using a confocal microscope equipped with a 40x oil immersion lens with a numerical aperture of 1.3 (Zeiss 5 Live, Jena, Germany). We used a 543 nm laser line for excitation of Alexa Fluor 555 (anti-vimentin). Emitted light was long-pass filtered at 560 nm. Image stacks had a spatial resolution of 0.31 × 0.31 × 0.31 *μ*m and were obtained with a field-of-view (X and Y directions) of 318 × 318 *μ*m extending up to 60 *μ*m into the myocardium (Z direction). The Z-axis was parallel to the laser beam direction. An example of a 3D-stack with processed images is illustrated in Fig 1 (left panel). The sampling in z-direction could have been reduced based on the Nyquist-Shannon theorem [47, 48]. However, as in all of our other microscopic studies we chose not to do so to simplify subsequent processing and visualization.

The WGA labelling marked the interstitial space and cell membranes in the samples. For the process of identifying PK cells and working myocytes the tubular system (T-system) was examined. A sparse T-system is present in PK fibres while a dense T-system is a feature of ventricular working myocytes [49].

### Geometric Reconstruction and Structural Analysis

Each of the 2D images from the 3D stacks was pre-processed to improve the segmentation step using previously developed methods [26, 50, 51]. Firstly, the iterative Richardson-Lucy deconvolution algorithm was applied to all images using a measured point spread function. Secondly, background signals were removed and depth-dependent attenuation corrected to minimize artifacts due to bleaching, scattering and absorption, thus reducing issues with thresholding and similar segmentation approaches. Signal-to-noise ratio (SNR) of each image stack was measured as described previously [34] and the ones with a SNR below 3 were rejected. The PK fibres, i.e. paths of connected PK cells, were manually segmented from the different 3D image stacks. Automatic segmentation techniques were not suitable for the accurate extraction of the PK network trajectories due to the varying levels of diffusion of the WGA in the tissue in different images. Therefore, we manually located landmarks along the PK pathways in all 3D image stacks at furcation points. The 3D Slicer software [52] (www.slicer.org) was used to identify the bifurcation points. Spatial coordinates of the landmarks were stored and were subsequently connected with straight lines, forming the fibre centrelines. Fig 1 (right panel) illustrates the 3D reconstruction of several PK strands together with the corresponding centrelines in red. These centrelines were then used to quantitatively analyse the PK network morphology (angles of furcations and complexity of network). In-house software developed using Matlab R2013b (MathWorks, Natick, MA) was subsequently used to reconstruct the PK pathways. A set of 10 stacks was selected to perform a full 3D segmentation and characterise the different cell shapes. A painting tool available in the software 3D Slicer allowed segmenting in 3D each cell across the stacks. Each cell was represented by a triangular closed surface mesh. The morphology of the reconstructed PK cells was quantitatively characterised (3D lengths, surface, volume) to explore possible different geometrical configurations. For the measurements of length and the diameters in tubular cells, multiple measurements were taken for each single cell and for each of its dimensions, which were subsequently averaged. Following the longitudinal axis of the cells, two perpendicular lengths were obtained, the height and the width of the cell relative to the endocardial plane. In star-like cells, the length was measured using as longitudinal length the longest trajectory between borders connected to neighbour cells. The area of the cells was calculated summing up the surface of each of the triangles that defined the boundary of the cell mesh. From the 3D surface representation, the volume was obtained using the divergence theorem.

### Analysis of Complexity of the PK Network

PK network characteristics were quantitatively analysed from the set of previously defined landmarks and centrelines along PK pathways. The parameters used to characterise the PK network were the following; i) branch lengths, considered as the set of connected PK cells without any furcation; ii) angles at furcation points, differentiating between acute (*α*) and obtuse angles (*β*). In general, at furcation points, cells are connected to two or more neighbouring cells forming branches with a ‘Y’ shape, which can be characterised by one acute and two obtuse angles. If all branches are arranged in a preferred direction, acute angles will be 0° whereas obtuse angles will be 180°. However, in networks that span widely, acute angles will be larger than 45° and obtuse angles 90°. These parameters were quantified in 3D by extraction from the 3D confocal microscopy imaging stacks, thus preserving the 3D nature of the PK network.

Quantitative measures based on graph theory were applied for characterising the complex PK networks extracted from each image stack. We considered state-of-the-art graph metrics [53] to estimate regional and global features of the PK network complexity, following the work of Rubinov and Sporns [54]. PK networks were then characterised by a set of connected nodes (i.e. furcation points) and links between these nodes (PK pathways) allowing the analysis of important features such as the shortest path length between two nodes of the network (their average often referred as to characteristic path length). The chosen metrics were then the following: i) the global efficiency (GE), defined as the averaged inverse shortest path length between all nodes of the network; ii) the local efficiency (LE) of a node, computed as the averaged inverse shortest path of all neighbours of the node; iii) and the degree, which refers to the number of neighbours a node has and estimates network infrastructure. Local efficiency, which is a measure of segregation, and global efficiency, which measures integration of the network, are combined to test for ‘small-world connectivity’, which is normally used to refer to networks with highly segregated modules and high number of inter-modular links (integration), such as in the case of the brain [53]. A small-world connectivity network will then be robust to the malfunctioning/removal of certain nodes connecting modules since it will be possible to find alternative small-distance paths between two given modules of the network. As in Achard and Bullmore [55], we used the following criteria for estimating small-world connectivity: *GE*_*latt*_ < *GE* < *GE*_*rand*_ and *LE*_*rand*_ < *LE* < *LE*_*latt*_, where *latt* is a lattice (regular) equivalent network and *rand* is a random equivalent network with the same nodal degree distribution and size as the original graph. These relations state that a small-world connectivity network will have a higher integration (links between different modules) than a lattice equivalent network but lower than a random one (based on GE). Furthermore, it will have more segregated modules than a random equivalent network but less than a lattice one (based on LE).

Additional graph metrics were used to characterise the PK network complexity, including: node betweenness (or node centrality), which is an indicator of a node’s centrality in a network, being equal to the number of shortest paths from all vertices to all others that pass through that node; and the assortativity coefficient, which estimates the correlation between the degrees of nodes on two opposite ends of a link and shows the preference for network’s nodes to attach to similar degree ones (indirectly measuring the vulnerability of a network to insult, i.e. broken links). For comparison purposes, the node betweenness is represented as a histogram for each network, with the number of shortest paths normalised by the maximum number of possible paths crossing each node of the network.

We refer to Boccaletti et al. [56] for more details about these measures. The Brain Connectivity Toolbox [54] was used to compute graph measures. Weighted global and local efficiencies were estimated by using the inverse distance of the edges of the network.

### Statistical Analysis

Data were expressed as mean standard deviation (SD) for all the measured lengths and angles. The circular statistics toolbox from Matlab CircStat [57] was employed to compute histograms, average and standard deviation of angles. Wherever appropriate, data were subjected to statistical analysis by one-way analysis of variance (ANOVA). A value of *p* < 0.05 was considered significant. Matlab software (MathWorks, Natick, MA) was used for the statistical analysis.

## Results

### Cell Identification and Morphological Analysis

**Fig 2:**
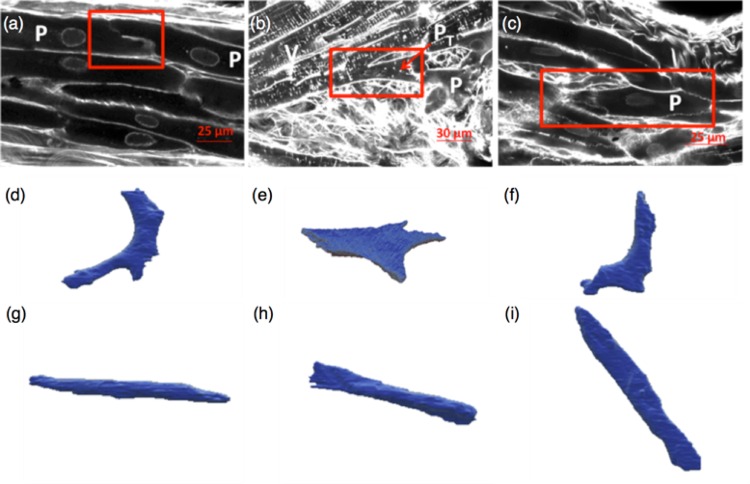
Confocal images with PK fibres and myocytes. (a) Example of a PK cell (P) with a sparse T-system and a PK end-to-end connection with a step profile (red square); (b) Example of a ventricular myocyte (V) with a regular dotted pattern T-system and a PK-myocyte connection (red square) where the PK cell, defined as transitional cell (P_T_), arrow) has a higher T-system density than regular PK cells; (c) Example of a star-like PK cell with absent T-system; (d)-(i) 3D reconstructions of PK cells with different morphologies, including star-like ((d)-(f)) and tubular ((g)-(i)) configurations.

Our results confirm a high T-system density in ventricular myocytes (labeled with V in Fig 2(b)) and almost absence of T-system in PK cells (labeled with P in Fig 2(a)–2(c)). This visual identification of the T-system was only possible due to the very high resolution of the acquired WGA-labelled images. Fig 2(a) shows the sparse T-system of the PK cells and their nuclei, in contrast to the regular dotted pattern of T-system in the working myocytes in Fig 2(b). The shape of PK cells varied significantly at furcation points, as compared to the regular tubular shape found in bundles and FRPS (Fig 2(d)–2(i)). The PK fibres at the junctions in general were more flattened and irregular, taking two of them to make a junction. We have referred to this type of cell as ‘star-like’ in this paper. These star-like PK cells, as seen in Fig 2(c), showed a lack of T-system. They are commonly connected to tubular PK cells and share with them a lateral connection. Five tubular and five star-like PK cells were segmented from the available data. Manual segmentation of individual PK cells was followed by its 3D surface reconstruction, in which we measured length, diameter, volume, surface area, and surface to volume ratio. Fig 2(d)–2(i) show three examples of each type of 3D reconstructed cell. Quantitative shape differences between the two types of PK cells are presented in Table 1. Although the sample was small, the similarity in volume and surface area between cell groups was remarkable.

**Table 1:** Geometrical measurements parameterizing the shape of individual PK cells.

Parameter	Tubular shape	Star-like shape
Length (*μm*)	119.76 ± 28.57	63.27 ± 6.27
Diameter sagittal (*μm*)	13.76 ± 3.60	19.70 ± 5.20
Diameter coronal (*μm*)	N/A	6.79 ± 1.51
Volume (*μm*^3^)	15756.32 ± 1573.40	15843.62 ± 6979.15
Surface area (*μm*^2^)	5604.27 ± 107.83	5648.84 ± 1892.67
Surface/volume (*μm*^−1^)	0.356	0.357

N/A: Not available

### Cellular Connections

Extracellular space was clearly labeled by WGA in the acquired images. Fig 2(a) shows a PK end-to-end connection with a step profile, as highlighted in the figure (red square). The collagen shielding of the PK fibres was only visible in the periphery, therefore we suggest that these cells are longitudinally connected. A PK-myocyte connection is shown inside the red square in Fig 2(b), where a single PK cell end-to-end connects with a ventricular myocyte. Nevertheless, the PK cell shown in this figure had a higher T-system density than regular PK cells (see arrow in Fig 2(b)). The backtrace of the PK strand in the proximal direction placed it at the endocardial insertion of a FRPS.

**Fig 3:**
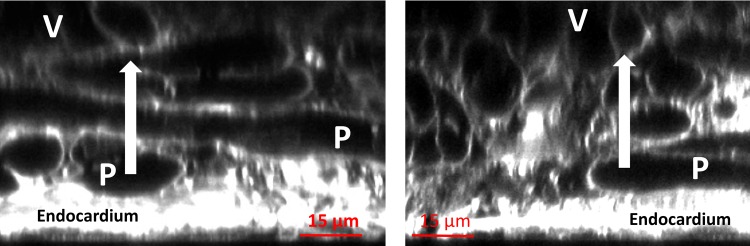
Longitudinal and cross sectional projections showing Purkinje-ventricle connections. Arrows indicate potential lateral connections of Purkinje (P) to ventricular myocytes (V). Potential transitional cells (P_T_) with different T-system configurations in between PK fibres and ventricular myocytes are also shown.

Visual inspection of longitudinal and cross sectional projections shown in Fig 3 suggests the existence of interconnections between PK cells and ventricular myocytes through lateral connections. This assumption is based on the close proximity between cell membranes and the absence of a clear separation in the extracellular labelling. The large white arrows in Fig 3 indicate the potential lateral connections of PK to ventricular myocytes. However, these observations cannot confirm whether these connections can transmit electrical impulses. Previous works [6] described a layer of transitional cells (P_T_) in between PK fibres and ventricular myocytes. In our imaging data we found cells with different T-system configurations in between PK fibres and ventricular myocytes (Fig 2(b)).

### Free Running Purkinje Strands (FRPS) and their Connections

**Fig 4:**
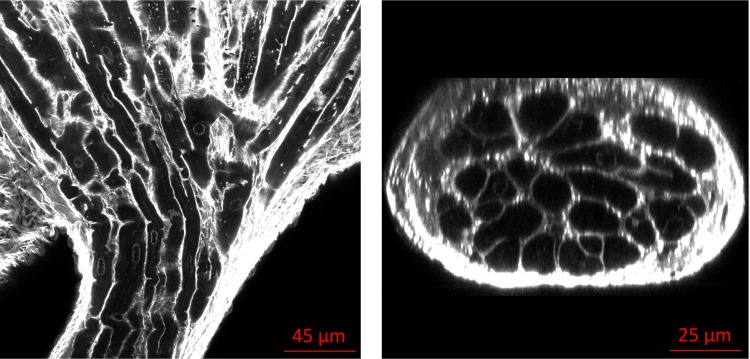
Example of Purkinje Free running strand of type I. Longitudinal (a) and cross sectional (b) views showing how PK fibres inside the strand are in contact transversally and share information through end-to-end connections longitudinally. As the strand reaches the endocardium, it spread out as a fan.

**Fig 5:**
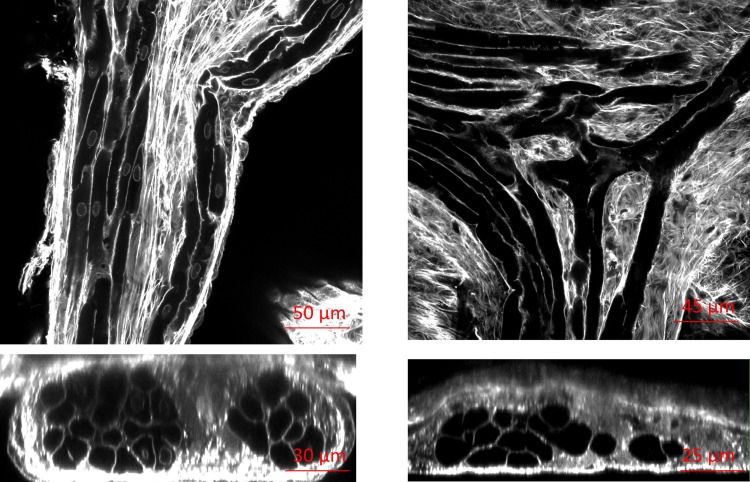
Examples of Purkinje Free running strands of type II. Longitudinal (a,b) and cross sectional (c,d) views showing how PK fibres are divided in different bundles inside the strand, separated by collagen. Left images (a,c) illustrate bundles inside a strand going to separate paths, while right images (b,d) depict how internal bundles exchange information creating a communication network.

The study of the FRPS led us to differentiate two types of fibre configurations. In type I all PK fibres inside the strand were in contact in the transversal section and shared the electrical information through end-to-end connections in the longitudinal direction. As the strand reached the endocardium it spread out as a fan (Fig 4). In the second type of FRPS fibres were divided into separate bundles inside the strand, using collagen for insulation. Fig 5(a) and 5(c) show how bundles inside a strand diverge going on their own separate paths as they approximate to the endocardium. Fig 5(b) and 5(d) show a case with a smaller strand type II penetrating through the endocardium where its internal bundles create an exchange of information by making a communication network.

### Angles and Branch Lengths of the PK Network

**Fig 6:**
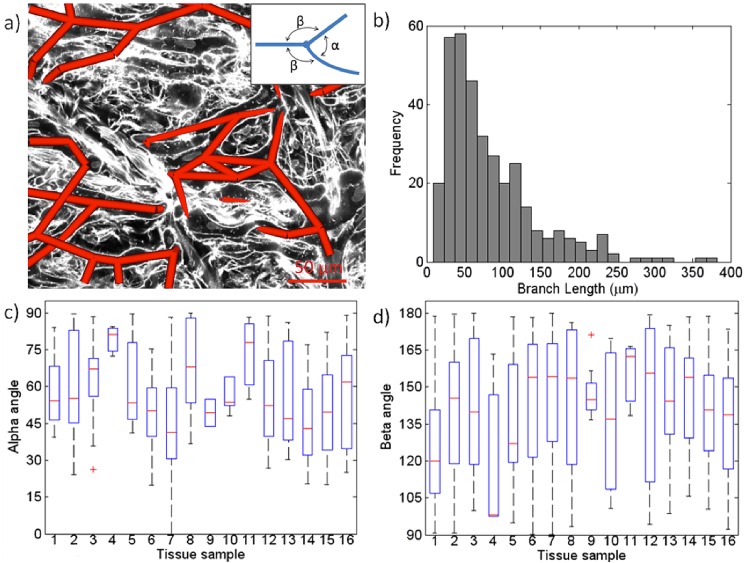
Quantitative measurements on segmented Purkinje branches obtained from 16 tissue stacks. (a) Sample slice from a stack imaged by confocal microscopy including corresponding segmented Purkinje cell as red tubes; (b) PK branch length histogram; Boxplots of angles between connected branches split in (c) acute angles (alpha) and (d) obtuse angles (beta) provided for each of the stacks analyzed. A graphical description of the angles is included in the detail of (a).

**Fig 7:**
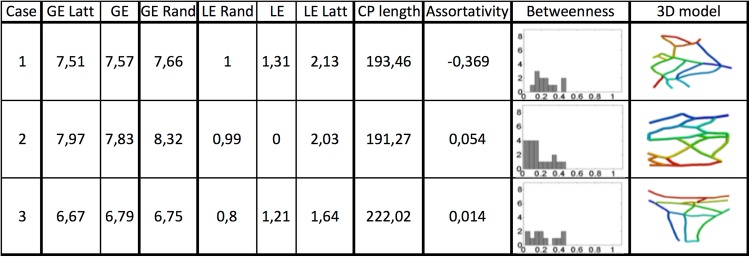
3D model examples of reconstructed PK networks and their corresponding graph theory measurements. G.E: global efficiency, G.E. Rand.: global efficiency random, G.E. Latt: global efficiency lattice, L.E.: local efficiency, L.E. Rand: local efficiency random, L.E: local efficiency lattice, C.P. Length: characteristic path length, Assort: Assortativity.

We extracted conduction pathways from 16 stacks of imaged tissue samples (see example in Fig 6(a)). Fig 7 illustrates the results of this segmentation for three representative cases (see Supplementary material for the description and views of all stacks). Branch lengths of the networks were computed as the length between two furcation points and plotted as a histogram in Fig 6(b). The mode in branch length distribution was at around 50 *μm*, with maximum lengths of about 300 *μm*. Its average length was of 84.6 *μm* and median of 65.6 *μm*. A concentration of short paths, due to several connected star-like cells, highlights the intricate communication among fibres at its microstructure. As expressed above, two angle populations were established based on their acute (*α*) or obtuse (*β*) nature at the furcations, as illustrated in Fig 6(a) (detail). Fig 6(c) and 6(d) show the angle distribution at each tissue sample for the *α* and *β* populations, respectively. We conducted a one-direction Anova test for each population of PK network branch angles, which determined that the values of the *α* population are not drawn from the same sample (p = 0.0013), and therefore they belong to two different populations. These acute angles presented a mean of 55.2° ± 20.6°, with values within the range [20°, 90°], as illustrated in Fig 6(c). In the case of obtuse angles (*β*) the Anova test showed these angles as to belong to the same population. The average of *β* angles was 140.19° ± 25.8°. From these values the selection of *β* angles seems to range the entire interval [90°, 180°]. The blue boxes in Fig 6(c) and 6(d) indicate the 25th and 75th percentiles, while the red and dashed lines represent the median and extreme values, respectively. On the other hand, the mean values for *β* angles are drawn from the same population (p = 0.1116).

### Complex Network Analysis

The complexity graph measurements performed on three examples of extracted conduction pathways is shown in Fig 7. (for all of them, see Supplementary material) The numerical order of the tissue samples (rows) corresponds to the order in which the data is presented in Fig 6(c) and 6(d). GE and LE were computed for a 100 random and 100 lattice equivalent networks of the same size and degree distribution (means presented in Fig 7). The criteria for small-world connectivity were only satisfied in one case (first sample, #1 in Fig 7). Eleven networks presented a negative assortativity coefficient, being the simplest non-redundant network (#4 in S1 Fig) the one showing the smallest assortativity with a value of -0.667, whereas the largest assortativity value was 0.066 (#16 in S1 Fig). With respect to the node betweenness, the shift to the right of its histograms in Fig 7 indicates the presence of highly travelled nodes, which means that a breakdown of one of them would likely change the path of many signals. On the other hand a histogram with the curve shifted to the left represents networks with more balanced distribution of paths. In our analysis, 12 out of 16 samples showed histograms shifted to the left, implying networks with redundant paths as observed in the path reconstructions in S1 Fig.

## Discussion

The main goal of our work was to characterise the 3D nature of the PK system. Previously, morphology of the conduction system and the PK cells was described based on only 2D images instead of 3D stacks, which makes it difficult to draw conclusions on the arrangement of the cells and the network. Due to the current settings of our imaging protocol and the large number of previous studies on the Purkinje system performed at macroscopic level, we focused specifically on the analysis of the Purkinje system at microscopic scale. Confocal microscopy images of the substructure of the PK network confirmed two types of cell morphology, as reported in [22], star-like and tubular PK cells. In the current work shape measurements were taken to characterise the two different cell populations. To our knowledge these PK cell measurements had not previously been taken from 3D reconstructions. Significant differences in cell lengths and diameters were established, although cells preserved a low variance in their surface to volume ratio (Table 1). These volume measurements are in good agreement to the values reported in [25], even though those were estimated on pig data: 5900 *μm*2 vs 5600 *μm*^2^ of surface area for [25] and for this work, respectively. On the other hand volume, surface/volume ratio indices and cell dimensions were substantially different ([25] vs this study): volume, 47k *μm*^3^ vs 16k *μm*^3^; surface/volume ratio, 0.15 *μm*^−1^ vs 0.35 *μm*^−1^; shorter in length but larger width in our study. Other researchers reported average dimensions of 164 × 35 *μm* in canine isolated PK cells [24] and 129 × 8 *μm* in rat isolated PK cells, which and 135 × 12 *μm* in rabbit PK cells [58], which are comparable to our value of 120 × 29 *μm* in rabbit. A possible explanation for some of these differences might be that measurements were mainly made on tubular cells. The morphological heterogeneity of PK cells raises the question of whether physiological differences are also present, being an interesting subject of further research. Differences in surface/volume ratios of cardiac working myocytes among mammalian species [59], and regional differences in myocyte dimensions have also been reported. A limited number of studies have been carried out to characterise the morphological and electrical behaviour of P_T_s at the PVJs. Ryu et al. [13] described P_T_ from histological stained samples, as rectangular shaped with a centrally located nuclei, as compared to round or oval shaped PK cells. In our current work P_T_ were positively identified in several data stacks by inspecting the integrity of the T-system on their membrane: PK cells lacked the T-system structure therefore showing smooth continuous membranes; on the contrary myocardium cells usually had a dense regular pattern of dots on the membrane representing the invaginations of the T-system; finally P_T_ cells showed a more sparse membrane dotted pattern (Fig 3). Doubts have been cast as to the existence of these cells at PVJs across all species, although the presence of transitional cells has been confirmed at many other parts of the cardiac conduction system, specifically in areas of transition/connection to the working myocytes, as in the case of the sinus node, the AV node or the His bundle [33, 60]. It has been reported in the literature [8] that the connection between PK fibres and working myocytes occurs in either of the two following ways: i) through a 2D interface made of P_T_ that separates the PK fibre from the myocardium, and ii) through a funnel connection in which the narrow side is a PK fibre to which muscle fibres connect progressively. In canine hearts Rawling et al. [7] described the electrical properties of transitional cells, and their coupling to PK and myocardial cells, which supported the idea of punctual or funnel PVJ connections. In an electrical analysis of the PVJs they indicated that at some locations multiple PK activation signals contribute to the junction process while others appear to be substantially uncoupled from neighbouring PK cell groups and the underlying transitional cells. This is contrary to the hypothesis of a 2D interconnection sheet (i.e. 2D interface) that connects PK and myocardium at many locations. The analysis of 3D confocal microscopy imaging data acquired on rabbits in this work suggests the presence of these two types of Purkinje-myocardium connections (i.e., 2D interface and funnel ones). The employed criteria were solely based on the proximity between cells and lack of collagen fibres in between them. We hypothesised that the connection among PVJs with 2D interfaces (Fig 3) is done through lateral connections of gap junctions. In different species connexins Cx43 and Cx40 have been found expressed on the sides of ventricular myocytes and PK fibres, respectively, although in much less density than at the cell ends. In [33] it was observed in mouse that Cx40 and Cx45 are co-expressed both in PK myocytes and in the underlying transitional cells, suggesting that Cx45 plays an important role in connecting up the Cx40/Cx43 PK myocyte compartment to the Cx43-expressing working ventricle via the transitional cells. Other structures of the cardiac conduction system such as FRPS were more easily identified. These structures are defined as cords made of a PK fibre bundle encased in connective tissue (i.e., collagen). They electrically link different parts of the endocardium [20]. False tendons have also been proven to carry PK fibres on the inside [5, 6]. Our study shows for the first time two different configurations of the PK fibres within the strand: i) type I strands, where all cells are in contact, therefore sharing electrical information (as can be observed in Fig 4); and ii) type II strands where two separated bundles run in parallel, with limited information exchange (Fig 5). Given the field of view of the available stacks of images we were not able to trace the strands to their origin. An intuitive approach at understanding the role of furcations points observed in these networks is to hypothesise that its main function is to combine electrical information by connecting PK fibres coming from different ends. We also hypothesise that type II strands might be the result of multiple strands coming together but without sharing conductive material. A possible explanation for maintaining electrical information segregated could be for safety issues; for instance if strand function is compromised at one end of the network (e.g. due to ischemia) the other ends can still receive electrical stimuli. The branch length pattern was found to follow an exponential distribution, in agreement with results previously reported for sheep [46], calf and lamb hearts [21] at the macroscopic level. The average lengths measured in these previous works cannot be strictly used for comparison given that the resolution of their images was substantially lower and studied species were also different. Nevertheless it is worthy to note that the branch length distribution provided by Ijiri et al. [46] has a range from 0 to 300 *μm* and an average length of 130 *μm*, whilst we have measured 84.6 *μm* in the present study on rabbits. With regard to the angles between branches, in [21] angles of 60.0° ± 40° were reported from macroscopic observations for acute angles (*α*), which are comparable to 55.2° ± 20.6° obtained at microscopic level. For obtuse angles (*β*), macroscopic observations show a range of 160° ± 20°, whereas microscopic measurements show a wider range of 140.19° ± 25.8°. In the complex network analysis based on graph theory measurements, we made several observations. The majority of the extracted PK networks did not fulfil the small-world connectivity criterion (just one out of 16). This means that the PK networks studied at this resolution did not show high modularity (clusters of nodes highly interconnected between them), being quite sensitive to any possible insult to the network. This is in contrast to the behaviour of other networks in the human body such as the brain, where the small-world connectivity criterion is fulfilled [53]. Further research is still needed to study if small-world connectivity is fulfilled in PK networks at more macroscopic resolutions and with larger fields of view. Eleven out of 16 of the analysed networks (69%) showed negative values of the assortativity coefficient, thus being vulnerable to insult (malfunctioning of a node or link), which correlates with the absence of small-world connectivity. Negative assortativity is a property that share biological networks in general, since high degree nodes such as star-like cells tend to join low degree nodes, such as tubular cells. When visually inspecting the reconstructed PK networks, one could easily identify four very simple networks (rows 4, 9, 10, 11 in S1 Fig), corresponding to non-plexus regions, which have the largest negative assortativity values. When ignoring simple networks in the study, there were a lower percentage of negative assortativity cases (58%). The low robustness to malfunctioning of some nodes in these networks could cause local microscopic conduction blocks due to alterations of network properties due to ischemia or pharmacological treatment among other factors. However, the PK network redundant structure observed at macroscopic level [12, 21] might compensate microscopic malfunctions. The betweenness measurement in the network analysis complemented the assortativity coefficient by providing local information about the relevance of a given node within the network. In our analysis, 12 out of 16 samples showed histograms shifted to the left, which involves a balanced distribution of PK network without critical hubs. This increases the safety factor of the network as a transmitter of the electrical impulse in case of a local damage of the structure. In addition, it is important to point out that in a PK network the electrical impulse does not travel only through the shortest paths, and that a damage or block in a section of the system, will only delay the impulse, but will not block it completely. The differentiation between PK and other myocardial cells was based on the high density of the T-system in working myocardial cells and its absence in PK ones. The use of WGA to identify the outer cell membrane, T-system and extracellular space has been established in a number of studies [61, 62]. While this study was based on confocal microscopy, other authors have used a variety of techniques for similar purposes, such as light microscopy [8], scanning and transmission electron microscopy [22, 60, 63], and confocal microscopy with lipophilic fluorescent dye di-8-ANNEPS [25]. Recent imaging modalities such as two-photon and light sheet microscopy could also be used to acquire complementary information about cardiac tissue. The segmentation of PK cells from 3D stacks was mainly manual given the complexity of the 3D structures and the imperfections in the WGA labelling. Semi-automatic methods such as region growing techniques were not effective due to the number of discontinuities on the cell membrane, causing them to leak through into the extracellular space. Although several recent semi-automatic and automatic segmentation techniques in cell images have been proposed [51] manual techniques still remain the gold standard.

### Limitations of the study

Analysis of imaging PK cells is a complex task, which involves labor-intensive experiments and tedious manual image processing where many tissue samples need to be acquired in order to have some of them fully analysed. The results presented in this paper correspond to sparse tissue samples from different endocardial regions, instead of a single region, which prevented us to draw conclusions on local differences of the network. Another limitation was the lack of data to relate microscopic and macroscopic features in the same specimen, which will be needed for a more consistent comparison across scales. The hypothesis based on the existence of lateral connections on PVJs still needs further validation with Cx40-Cx43 labelling studies to confirm their presence in these areas of close proximity and lack of collagen. Finally, 10 datasets were processed to extract cell segmentations and then derive morphological indices due to the complexity of the task that was performed manually. The limited field of view of the acquired microscopic data resulted in simple networks with a small number of nodes and links, unlike dense networks (e.g. brain connectome) where graph metrics are usually applied, making some of the complexity network estimates difficult to interpret.

## Conclusions

This study inspects the morphology of PK cells in 3D, revealing the different types of cell shapes, that conform the large network infrastructure of the cardiac conduction system at distal sections. The configurations and pathways formed by the PK system show a high (but heterogeneous) degree of robustness at the microscopic level as obtained after performing a network analysis. These results might help building more realistic computational PK systems at high resolution levels including different cell configurations (e.g. star- and tubular-shaped cells). In addition, better knowledge on the organisation of the network might help in understanding the collateral effects that several treatments such as radio-frequency ablation might have when the PK system is disrupted locally. For instance, isolating regions of the Purkinje network with functional PMJs could give rise to ectopic activity within the PK isles due to the automaticity of the Purkinje cells in certain scenarios. Another side effect could be due to the disruption of the PMJs that help to propagate retrogradely electrical signals generated from a pacemaker. All these hypotheses require further studies in the future.

## Supporting Information

S1 Fig 3D model of reconstructed PK networks and their corresponding graph theory measurements for 16 tissue stacks. G.E: global efficiency, G.E. Rand.: global efficiency random, G.E. Latt: global efficiency lattice, L.E.: local efficiency, L.E. Rand: local efficiency random, L.E: local efficiency lattice, C.P. Length: characteristic path length, Assort: Assortativity.
(TIFF)
