# Supplementary figures and images for: Analysis of Microstructure of the Cardiac Conduction System Based on Three-Dimensional Confocal Microscopy
Source: PLoS One. 2016 Oct 7;11(10):e0164093. doi: 10.1371/journal.pone.0164093 (PMC5055359; doi:10.1371/journal.pone.0164093)

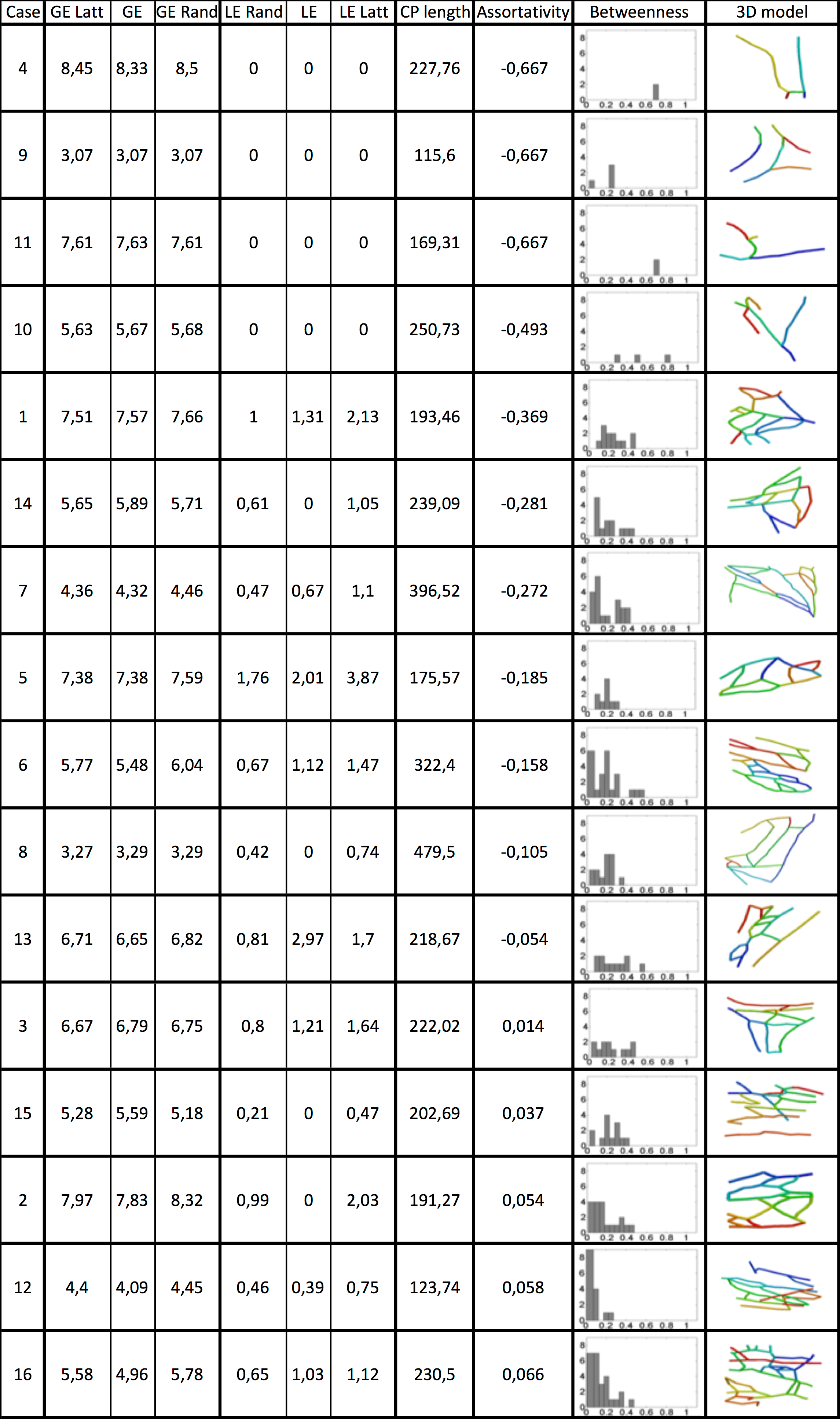

Supplement: S1 Fig — G.E: global efficiency, G.E. Rand.: global efficiency random, G.E. Latt: global efficiency lattice, L.E.: local efficiency, L.E. Rand: local efficiency random, L.E: local efficiency lattice, C.P. Length: characteristic path length, Assort: Assortativity. (TIFF) [file pone.0164093.s001.tiff]
